# Association Between Gastrointestinal Symptoms and Anxiety Levels in Patients with Functional Dyspepsia

**DOI:** 10.7759/cureus.84810

**Published:** 2025-05-25

**Authors:** Vaisnavy Govindasamy, Yashar Mashayekhi, Marium Nadeem Khan, Oluwaronke Joan Olakunori-Ovaga, Naresh Kumar, Mahnoor Zafar, Sufyan Mustafa, Muhammad Soman, Muhammad Bilal Akbar, Timsal Arif, Asiya Karim, Muhammad Murad Murtaza, Sher Bano

**Affiliations:** 1 Medicine, South Tees NHS Trust, Middlesbrough, GBR; 2 Medicine, Leicester University Hospital, Leicester, GBR; 3 Department of Medicine, Shifa College of Medicine, Islamabad, PAK; 4 Public Health, University of Sunderland, Sunderland, GBR; 5 General Medicine, Liaquat University of Medical and Health Sciences, Hyderabad, PAK; 6 Internal Medicine, Rashid Hospital, Islamabad, PAK; 7 General Medicine, Dow University of Health Sciences, Karachi, PAK; 8 Peadiatrics, Combined Military Hospital (CMH) Medical College, Kharian, PAK; 9 Psychiatry, Tallaght University Hospital, Dublin, IRL; 10 Internal Medicine, Combined Military Hospital (CMH) Medical College, Kharian, PAK; 11 Medicine, Lahore Medical and Dental College, Lahore, PAK; 12 General Medicine, Combined Military Hospital (CMH) Lahore Medical College and Institute of Dentistry, Lahore, PAK; 13 Clinical Psycology, NeuroWave Research Center, Islamabad, PAK

**Keywords:** anxiety, functional dyspepsia, gastrointestinal symptoms, psychological distress, quality of life

## Abstract

Background

Functional dyspepsia (FD) is a common gastrointestinal disorder characterized by chronic upper abdominal symptoms, such as epigastric pain, burning, postprandial fullness, or early satiation, in the absence of structural abnormalities, as defined by the Rome IV criteria. Anxiety, together with other psychological factors, is now understood to intensify symptoms while degrading the quality of life for patients with FD.

Methods

A cross-sectional correlational study involving 300 FD participants was performed in a gastroenterology outpatient clinic located in Islamabad. Participants were diagnosed with FD using the Rome IV criteria. The research collected data through self-report scales to assess gastrointestinal symptoms, anxiety levels, and quality of life. Generalized Anxiety Disorder-7 (GAD-7) measured anxiety symptoms while patients were evaluated for gastrointestinal symptoms through the Gastrointestinal Symptom Rating Scale (GSRS) and Short Form Nepean Dyspepsia Index (SF-NDI). Statistical analyses included Pearson correlation, t-tests, one-way ANOVA, chi-square tests, and multiple regression.

Results

Out of the 300 participants, 153 were females (51%), 118 were males (39%), 29 did not disclose their gender (10%), and 111 were aged between 18 and 25 years (37%). A majority of self-administer drugs for FD, with 59% (n = 178) of the respondents and 56% (n = 167) having a diagnosed psychological condition. Pearson correlation analyses revealed that GSRS and SF-NDI correlation coefficient was significant and equal to 0.789 and the correlation coefficient of GSRS and GAD-7 was 0.703 the correlation coefficient of SF-NDI and GAD-7 scores was 0.631 and p-value was significant < 0.001 for all the coefficients while checking the association between gastrointestinal and dyspeptic symptoms and quality of life as well as anxiety. Moreover, participants who used medications had significantly higher perceived symptom severity and anxiety levels than participants who did not use medication (t = 7.47, p < 0.001) with a mean GSRS score of 41.8 (SD = 9.3, n = 177) and a mean GAD-7 score of 21.8 (SD = 3.8) for the medication users, and a mean GSRS score of 35.3 (SD = 16.4, n = 123) and a mean GAD-7 score of 16.9 (SD = 7). In a multiple regression model, both gastrointestinal symptoms with a coefficient of 0.543 (t = 3.218; p≤ 0.01) and dyspeptic symptoms with a coefficient of 0.203 (t = 1.695; p≤ 0.01) were correlated with anxiety, so it can be assumed that severity of the symptoms has an impact on psychological well-being.

Conclusion

Findings indicate a significant association between gastrointestinal symptoms and anxiety in FD patients, highlighting the need for integrated gastroenterological and psychological care. FD physicians should provide routine anxiety screenings as part of their patient management to enhance both treatment effects and patient life quality.

## Introduction

Functional dyspepsia (FD) is defined by postprandial fullness, early satiety, epigastric pain, and burning or epigastric discomfort. Also, it is common to have other symptoms above the diaphragm, namely nausea, burping, and obstruction in the upper abdomen. It is diagnosed by the presence of these symptoms without any physical or biochemical cause. FD affects life quality seriously and results from abnormal gastric emptying and visceral hypersensitivity [1,2]. People suffering from FD make up about 16% of the healthy population, while the condition is frequently associated with anxiety disorders. The pathophysiology of FD is caused by dysregulation of the gut-brain axis, central nervous system (CNS) dysfunction, and consequently, the condition manifests chronically with fluctuating symptoms and limited treatment options [3]. Two central mechanisms that occur are impaired gastric accommodation, the decreased capacity of the stomach to relax after eating, occurring in about 40% of FD cases, and visceral hypersensitivity, increased sensitivity to gastrointestinal (GI) stimuli produced by aberrant sensory processing and afferent nerve activity [4,5]. FD affects 5-11% of the population with symptoms of epigastric pain or burning along with early satiety and postprandial fullness when endoscopy does not show any identifiable abnormalities [6,7].

FD is closely associated with higher rates of depression, and the same pattern exists with anxiety and neuroticism, particularly when FD continues to persist. Patient quality of life (QoL) and emotional distress, along with food intake, suffer major detrimental effects from psychological factors that need identification as part of improving treatment success [8,9].

FD reduces gastric accommodation capacity, while state anxiety, together with anxiety disorders, acts as an influencing factor. FD patients displayed delayed gastric emptying and lower state anxiety associated with accommodation changes, which demonstrated sophisticated behavioral and sensorimotor dysfunctions related to FD [10].

Anxiety disorders strongly influence GI problems like nausea, yet depression shows less impact on such symptoms. The presence of primary care GI symptoms closely relates to depression and anxiety symptoms to the extent that each additional GI symptom leads patients toward receiving an anxiety disorder diagnosis [11,12]. The specific anxiety affecting the gastrointestinal system (GSA), defined as heightened anxiety with accompanying hypervigilance, demonstrates strong links to worsened irritable bowel syndrome (IBS) symptom intensity, together with reduced QoL for such patients. GSA, along with general anxiety and depression, serves as a key predictor of both symptom severity and mental QoL [13]. The risk of depression develops in people with GI disorders at a rate of 7%, while anxiety develops at 8.8%. Research findings confirm that GI disorders should be part of the analysis that assesses their effects on mental health [14].

Evaluating how GI symptoms relate to anxiety levels in FD patients leads to the development of successful multi-specialty treatment approaches. This research utilizes a cross-sectional design to investigate how serious GI symptoms relate to anxiety levels in people with FD in South Asian medical settings.

FD is a frequently occurring GI condition that causes ongoing upper stomach pain, but medical tests fail to detect structural abnormalities. Despite being a non-organic condition, FD seriously affects patients’ QoL and requires significant healthcare system resources. Studies reveal that functional GI disorders manifest as a complex relationship between psychological events and physiological factors, with anxiety increasing symptom perception and exacerbation.

FD patients exhibit higher anxiety levels, and this psychological state affects their gastric movement as well as their sensitive organs and pain signal processing in their brains. The importance of the gut-brain axis is highlighted by this connection because it shows the two-way neurological communication between the brain and gut. Few studies have explored the relationship between anxiety and FD symptom intensity alongside life quality.

The research is intended to evaluate the relationship between GI symptoms, together with anxiety indicators, along with quality-of-life results for individuals with FD by applying validated assessment tools (Gastrointestinal Symptom Rating Scale (GSRS), Generalized Anxiety Disorder-7 (GAD-7), and Short-Form Nepean Dyspepsia Index (SF-NDI)). The widely validated GAD-7 assessment tool enables evaluations of anxiety symptoms both clinically and in research studies, including functional GI disorders. The validated assessment tools of the GSRS and SF-NDI excel in measuring FD’s impact on GI symptoms with their measurement of dyspepsia-related QoL. Research of these associations helps create multidimensional therapeutic approaches that combine psychological and GI treatment methods to promote better patient results.

Objectives

The primary aim of the study will be to assess the association between GI symptoms and anxiety in patients with FD defined according to the Rome IV criteria [15]. We hypothesize that greater GI symptom severity is associated with higher anxiety scores.

Secondary outcomes are to measure the effect of FD on the patients’ QoL using the SF-NDI and to measure frequency and severity of symptoms using the GSRS before and after the intervention. Further, the study seeks to establish the role of anxiety as assessed by the Generalized Anxiety Disorder-7 (GAD-7) scale as a predictor of both GI and QoL symptoms in FD.

## Materials and methods

Study design

The research adopts a cross-sectional correlational framework to evaluate the relationship between GI symptoms and anxiety levels in FD patients. A single data collection period will take place with patients who seek gastroenterology outpatient care. The existing design enables researchers to determine patterns between variables without changing any actual conditions. Such a method delivers an efficient assessment of psychological and physical health relationships for clinical settings. The study obtained approval from the institutional review board, NeuroWave Research Center, Islamabad, Pakistan, with IRB-2025-0079, together with participant consent, before moving forward with data collection based on a specific protocol. The participants received an explanation about the research objectives, and volunteers received explicit confirmation regarding their free choice to participate.

Study setting and duration

The research took place within the gastroenterology outpatient department of a hospital located in Islamabad, Pakistan. Patients underwent data collection from February to April 2025. The enrolled patients were diagnosed according to Rome IV criteria with FD as determined by purposive non-probability sampling.

Participants

The sample size was determined by the WHO sample size formula for proportions with a population proportion of 0.90, a 95% confidence level, and a 5% margin of error [16]. According to these parameters, it was deemed necessary to achieve at least 300 participants in the study sample for representation and statistical purposes. The study involved adult subjects who had FD as a clinically confirmed diagnosis within the gastroenterology outpatient department based on Rome IV criteria [15]. The study selected participants who were at least 18 years old, along with the requirement to give informed consent and absent any GI organic disorders or major psychiatric illnesses. Patients previously diagnosed with peptic ulcer disease, GERD, or any other form of GI malignancy were excluded from the study to make sure that they had no other disease that would contribute to their dyspeptic symptoms. Furthermore, patients receiving psychotropic medicines or those diagnosed with anxiety or depression and receiving active treatment were excluded from the study to eliminate confounding effects on the measures of anxiety, since these conditions and treatments could influence both anxiety and GI symptoms.

Data collection tools and procedure

This research study utilized three validated self-report instruments alongside a demographic information form that measured GI symptoms together with anxiety levels, along with QoL.

The GSRS evaluated the frequency and intensity of GI symptoms involving indigestion, abdominal pain, reflux, diarrhea, and constipation. Jan Svedlund, together with Ingemar Sjödin and Gerhard Dotevall, developed the GSRS in 1988. The scale measures GI symptoms through a Likert scale assessment system from 1 (no symptoms) to 7 (severe symptoms) for conditions of constipation, bloating, abdominal pain, diarrhea, and indigestion. Clinical researchers have documented the GSRS scale reliability scores between 0.61 and 0.83 [17]. To evaluate patient QoL along physical and emotional domains, researchers used the Short Form Nepean Dyspepsia Index. The assessment tool contains five domains that measure both symptom intensity and its impact on day-to-day activities to effectively evaluate the vast consequences of FD on patients’ QoL. It was developed by Talley NJ, Verlinden M, and Jones M in 2001 to assess the impact of dyspepsia on health-related QoL. Research shows that the tool possesses strong psychometric properties, whose test-retest reliability has proven excellent at 0.90, as indicated by intraclass correlation coefficients. Respectively, Cronbach’s alpha indicated values between 0.83 and 0.88 for the subscales [18]. The Generalized Anxiety Disorder 7-item scale served to evaluate participant anxiety levels through scale administration. The GAD-7 serves as a self-report measure that Spitzer and colleagues developed in 2006 with seven items rated through a 4-point scale between 0 (not at all) and 3 (nearly every day). Participants obtain total scores from 0 to 21 through the scale, which demonstrates anxiety symptom intensity. The GAD-7 shows excellent internal consistency through Cronbach’s alpha rating of 0.92 and test-retest reliability using an intraclass correlation coefficient of 0.83 [19]. The GAD-7 scale evaluates anxiety to detect anxiety symptoms. GAD-7 serves as a validated screening instrument but does not provide sufficient criteria for psychiatric diagnosis. Professionals who conduct MINIs together with Mental Status Examinations need to diagnose psychiatric conditions. The study explicitly acknowledges its restrictions in this area. In addition to these scales, a demographic information sheet was utilized to further collect data about participants’ age, sex, marital status, education, and medical history (Table 1).

**Table 1:** Instruments Used

Scale	Purpose	Developers	Structure & Scoring	Reliability
Gastrointestinal Symptom Rating Scale (GSRS)	Assesses frequency and intensity of GI symptoms (e.g., indigestion, abdominal pain, diarrhea)	Svedlund et al. (1988) [17]	15 items, 7-point Likert scale (1 = no symptoms, 7 = severe symptoms)	Cronbach’s alpha: 0.61–0.83
Short Form Nepean Dyspepsia Index (SF-NDI)	Evaluates the quality of life across five domains affected by dyspepsia	Talley et al. (2001) [18]	10 items covering physical and emotional impacts	Cronbach’s alpha: 0.83–0.88; Test-retest ICC: 0.90
Generalized Anxiety Disorder-7 (GAD-7)	Screens for anxiety severity	Spitzer et al. (2006) [19]	7 items, 4-point scale (0 = not at all to 3 = nearly every day); total score range: 0–21	Cronbach’s alpha: 0.92; Test-retest ICC: 0.83

Participants who accepted the request completed independent questionnaires consisting of the GSRS SF-NDI and GAD-7 in private facilities to protect their confidentiality. A sufficient duration was designated to complete the forms, and the process required 10-15 minutes per participant. After that, all the questionnaires were checked by the researchers themselves with the purpose of completeness and accuracy. Based on this, any questionnaires with missing responses amounting to more than 10% of the scale items were omitted from analysis. Whenever the amount of missingness amounted to more than 10% of total items, the mean imputation was applied to the scale. Information obtained from the completed data was archived and later transferred into statistical analysis software to analyze data and to maintain the anonymity of the participants.

Statistical analysis

The analysis of data was conducted through IBM SPSS Statistics version 26 (IBM Corp., Armonk, NY, USA). The research used descriptive statistics to analyze demographic factors, including participant age together with gender status and marital situation and attained education and current occupation, and household income. To determine the suitability of applying t-tests and ANOVA, normality of the data was checked using the Shapiro-Wilk test, histograms, Q-Q plots, and skewness & kurtosis coefficients. Therefore, to confirm the homoscedasticity, the results of the Levene's test were also checked. The research used Pearson correlation to determine associations between scores from three measurement scales, including the GSRS and the Nepean Dyspepsia Index, and the Generalized Anxiety Disorder. The research used independent samples t-tests and one-way ANOVAs for comparing the variables between different groups, including medication status, age categories, and gender. The study used chi-square tests to detect relationships between FD symptoms and psychological conditions, and demographic variables, and multiple linear regression analysis was utilized to examine the predictive value of the GI symptom severity (GSRS) and quality-of-life impairment (SF-NDI) in terms of levels of anxiety (GAD-7 scores). The regression model was selected to determine the independent contribution that each predictor variable makes to the severity of anxiety while adjusting for possible confounders. Assumptions of linearity, homoscedasticity, independence of errors, and multicollinearity were tested and confirmed before interpreting the regression model. All statistical procedures used a significant value of p < 0.05.

## Results

Table 2 shows the participant demographics from the study sample, which included 300 individuals. The research sample contained 111 participants who fell between 18 and 25 years old and represented 37% of the total, and they were the largest group. Additionally, 56 participants (19%) among 26 to 35 years old and 59 participants, comprising 20%, within 36 to 45 years were also part of the study. The research data showed 38 participants (13%) from the under-18 age group, while such groups aged 46-55 years and 56+ years included 19 (6%) participants and 17 (6%) participants, respectively.

**Table 2:** Demographic Characteristics of Participants (N=300) f: frequency; %: percentage

Variable	f	%
Age		
Under 18 years	38	13
18-25 years	111	37
26-35 years	56	19
36-45 years	59	20
46-55 years	19	6
56 years or above	17	6
Gender		
Male	118	39
Female	153	51
Prefer not to say	29	10
Marital status		
Single	96	32
Married	129	43
Divorced	59	20
Widowed	16	5
Educational level		
No formal education	37	12
Primary school	74	25
Secondary school	87	29
Intermediate (FA/FSC)	71	24
Bachelors	20	7
Masters	11	4
Occupation		
Student	100	33
Employed	116	39
Unemployed	65	22
Retired	19	6
Monthly income		
Below 25,000	53	18
25000-50,000	81	27
51,000-100,000	130	43
Above 100,000	36	12
Duration of functional dyspepsia symptoms		
Less than 6 months	70	23
6 months- 1 year	100	33
1-3 years	61	20
More than 3 years	26	9
Prefer not to tell	43	14
Medication for gastrointestinal symptoms		
Yes	178	59
No	122	41
Diagnosed psychological conditions		
Yes	167	56
No	94	31
Prefer not to say	39	13

Among the participants, 153 (51%) self-identified as female, whereas 118 (39%) identified as male, and 29 (10%) did not tell their gender. Of the participants, 129 (43%) were married and 96 (32%) chose single status, while 59 (20%) were divorced, and 16 (5%) had lost their partners due to death.

Secondary school emerged as the highest educational level chosen by participants, with 87 cases (29%), while 71 respondents (24%) selected intermediate (FA/FSC), and 74 people (25%) finished primary school. The data shows that 37 participants (12%) had no formal education, 20 (7%) and 11 (4%) had bachelor’s and master’s degrees, respectively.

Out of 300 total participants, 116 people (39%) worked, 100 (33%) went to school, 65 (22%) did not have a job, and 19 (6%) were in retirement. Among survey participants, 130 individuals (43%) earned salaries between 51,000-100,000, while 81 participants (27%) received 25,000-50,000, and 53 (18%) earned below 25,000, and 36 people (12%) earned more than 100,000 each month.

A total of 100 patients (33%) experienced FD symptoms between six months and one year while 70 participants (23%) presented symptoms for a shorter period than six months and 61 patients (20%) had 1-3 years of symptoms followed by 26 patients (9%) who reported symptoms longer than three years and 43 patients (14%) refused to share this information.

A total of 178 respondents (59%) took GI medications, while 122 people (41%) did not require them. Of the total participants, 167 (56%) had been diagnosed with psychological conditions, whereas 94 (31%) had not received any diagnosis, and 39 (13%) declined to give their information.

Table 3 reports on the intercorrelations between the study variables. The GSRS exhibited a very strong statistical connection with the Nepean Dyspepsia Index through their correlation value of r = 0.789 (p < 0.001), which verified that GI symptoms match reported dyspepsia severity levels. The GAD scores demonstrated statistical correlation with both the GSRS (r = 0.703, p < 0.001) and the Nepean Dyspepsia Index (r = 0.631, p < 0.001). The study demonstrates that heightened anxiety creates a strong relationship with the occurrence of GI symptoms and worsening dyspepsia severity within this group of patients.

**Table 3:** Intercorrelations between the Study Variables *p<0.05 **p<0.001 considered significant; correlation= Pearson Correlation

Variable	The Gastrointestinal Symptom Rating Scale	Nepean Dyspepsia Index	Generalized Anxiety Disorder
The Gastrointestinal Symptom Rating Scale	-	0.789^**^	0.703^**^
Nepean Dyspepsia Index	0.789^**^	-	0.631^**^
Generalized Anxiety Disorder	0.703^**^	0.631^**^	-

Table 4 shows psychological and physical health measurement outcomes between participants on FD medication and those on no medication. Participants on GI medication reported significantly higher GSRS scores (M = 41.8, SD = 9.3) than non-medicated participants (M = 35.3, SD = 16.4), t(df) = 4.380, p < 0.001, with a moderate effect size (Cohen’s d = 0.49), reflecting more severe symptoms in the GI region.

**Table 4:** Comparison among Variables (Medication for Functional Dyspepsia Symptoms) M: mean; SD: standard deviation; LL: lower limit; UL: upper limit; Cl: confidence interval; independent t-test

Variable	Yes (N=178)	No (N=122)	t	P	Cl 95%		Cohen’s D
	M±SD	M±SD			LL	UL	
The Gastrointestinal Symptom Rating Scale	41.8±9.3	35.3±16.4	4.380	<0.001	3.594	9.457	0.49
Nepean Dyspepsia Index	31.2±8.6	26.3±14.2	3.691	<0.001	2.276	7.474	0.42
Generalized Anxiety Disorder	21.8±3.8	16.9±7.2	7.700	<0.001	3.661	6.175	0.85

People who used medication showed increased Nepean Dyspepsia Index mean dyspepsia severity of 31.2 ± 8.6, while non-medicated participants scored 26.3 ± 14.2 on this scale. This significant difference (t = 3.691, p = 0.000) produced a moderate effect size (Cohen’s d = 0.42). Research results indicated that those taking medication reported higher GAD scores, which amounted to 21.8 ± 3.8, than non-medicated participants who scored 16.9 ± 7.2, showing strong statistical significance (t = 7.700, p = 0.000) and a large effect size (Cohen’s d = 0.85). Medical treatment users showed more significant dyspepsia, together with GI symptoms, yet had increased rates of anxiety according to the study results.

Table 5 shows the average scores from different age groups reported on the GSRS and Nepean Dyspepsia Index, with the Generalized Anxiety Disorder Scale results. The GSRS produced its most excellent mean scores among people who belonged to the 26-35 years (43.8 ± 6.0), 46-55 years (43.9 ± 19.7), and 56 years or above (43.7 ± 12.5) age segments, reflecting more severe GI symptoms therein. The year’s age group displays the lowest average GSRS score of 29.9 ± 17.3 points. Research findings showed a significant statistical difference in GI symptoms based on age group (F (5,294) = 9.527, p < 0.05) with a sizable effect (η² = 0.40). Subject participants in the age range under 18 and 56 years or above registered the most severe symptoms with Nepean Dyspepsia Index scores of 32.4 ± 9.3 and 34.1 ± 12.6, respectively. The 36-45 years group scored the lowest at 22.5 ± 14.4. Dyspepsia symptom levels displayed significant differences across age groups based on an F value of 6.566 and an effect size of 0.100. Results showed that adults between 26 and 35 years old, together with individuals under 18 years of age, demonstrated the most significant levels of Generalized Anxiety Disorder (22.1 ± 3.8 and 21.1 ± 4.0). The 36-45 years age group demonstrated the minimum anxiety scores at 14.8 ± 7.7. Heightened levels of anxiety were observed in younger adults than middle-aged individuals, as indicated by results that demonstrated both high significance and large effect size (F = 13.805; η² = 0.190).

**Table 5:** Comparison of Variables (Age) M: mean; SD: standard deviation; F: F-ratio; η^2^: effect size; one-way ANOVA

Variable	Under 18 years (N=38)	18-25 years (N=111)	26-35 years (N=56)	36-45 years (N=59)	46-55 years (N=19)	56 years or above (N=17)	F (5,294)	η^2^
	M±SD	M±SD	M±SD	M±SD	M±SD	M±SD		
The Gastrointestinal Symptom Rating Scale	40.6±10.2	39.8±10.1	43.8±6.0	29.9±17.3	43.9±19.7	43.7±12.5	9.527	0.140
Nepean Dyspepsia Index	32.4±9.3	29.5±9.7	32.1±8.5	22.5±14.4	30.1±13.6	34.1±12.6	6.566	0.100
Generalized Anxiety Disorder	21.1±4.0	20.8±4.2	22.1±3.8	14.8±7.7	19.4±8.7	20.9±5.0	13.805	0.190

Table 6 shows an evaluation of GI symptoms and dyspepsia severity, together with generalized anxiety assessment data from male participants, female participants, and participants who did not disclose gender information. The GSRS yielded elevated mean scores of 41.6 ± 9.6 from male participants, whereas female participants scored 35.5 ± 14.3, and non-disclosing participants scored 48.7 ± 11.5. Research findings show significant GI symptom reporting differences between genders (F(2, 297) = 17.258, η² = 0.104), which indicates substantial gender effects. Participants who declined to reveal their gender section (not to say) experienced the most severe dyspepsia according to the Nepean Dyspepsia Index results, which revealed scores at 40.0 ± 10.6. In contrast, males scored 30.8 ± 8.8, and females scored 25.9 ± 12.0. The findings provided evidence that gender influences dyspepsia symptom severity since participants showed significant differences (F = 23.179) with an effect size that ranged between moderate and large (η² = 0.135). The responses of male participants exceeded those of female participants for Generalized Anxiety Disorder (GAD) (21.5 ± 4.3 compared to 18.3 ± 6.5), in addition to those who chose not to reveal their gender, scoring similarly to males (21.2 ± 6.4). A statistically significant difference between groups existed (F = 11.167) while possessing an effect size of (η² = 0.070). The results showed that gender made a substantial impact on GI distress and dyspepsia levels, together with anxiety, because non-disclosing participants maintained the highest scores for each measure.

**Table 6:** Comparison of Variables (Gender) M: mean; SD: standard deviation; F: F-ratio; η^2^: effect size; one-way ANOVA

Variable	Male (N=118)	Female (N=153)	Prefer not to say (N=29)	F (2,297)	η^2^
	M±SD	M±SD	M±SD		
The Gastrointestinal Symptom Rating Scale	41.6±9.6	35.5±14.3	48.7±11.5	17.258	0.104
Nepean Dyspepsia Index	30.8±8.8	25.9±12.0	40.0±10.6	23.179	0.135
Generalized Anxiety Disorder	21.5±4.3	18.3±6.5	21.2±6.4	11.167	0.070

Table 7 demonstrates how GI symptoms alongside dyspepsia severity levels predict GAD using multiple regression analysis. The constant value stood at 7.051, its 95% confidence interval spanned 5.542 to 8.560, and its standard error equaled 0.767. The GSRS was a powerful predictor of anxiety through its unstandardized coefficient (B) value of 0.247 at 0.188 and 0.306. The study revealed a strong positive relationship between the predictors through Dell’s standardized beta coefficient (β) with a standard error of 0.030. The analysis delivered significant results, which exceeded the p-value of 0.01. Analysis of the Nepean Dyspepsia Index revealed a relationship with anxiety that generated a B value of 0.105 (CI: 0.038 to 0.172 with 0.034 as the standard error and β of 0.203. The study indicates that GI symptoms, together with dyspepsia severity levels, serve as important anxiety predictors, but GI symptoms show a stronger predictive power. The research findings demonstrate the clear mental stress that accompanies problems in the digestive system.

**Table 7:** Multiple Regression for Study Variables constant: generalized anxiety disorder; B: coefficient; SE: standard error; β: standardized coefficient; LL: lower limit; UL: upper limit; Cl: confidence interval **p<0.01 considered significant.

Variable	B	95% Cl		SE	β	p
		LL	UL			
Constant	7.051	5.542	8.560	0.767	-	<0.001
The Gastrointestinal Symptom Rating Scale	0.247	0.188	0.306	0.030	0.543	<0.001
Nepean Dyspepsia Index	0.105	0.038	0.172	0.034	0.203	<0.001

Table 8 displays descriptive statistical information about age groups and gender distributions, together with FD symptoms alongside psychological conditions. It also includes chi-square (χ²) test results. The data analysis showed significant effects from the age-to-gender relationship (χ² = 37.6, p = 0.000) and the age-to-psychological conditions relationship (χ² = 42.9, p = 0.000). This indicates that the age distribution demonstrated significant differences between both genders’ groups and psychological profiles. Three age brackets were represented in the study: the 18-25 years group contained 111 participants, and the 26-35 years group and 36-45 years group, respectively, had 56 and 59 participants. The gender statistics across age groups showed uniform distribution, but some participants kept their gender information private. Gender distribution along with psychological conditions demonstrated significant statistical associations to FD symptom duration based on the chi-square test results (χ² = 18.0, p = 0.021, χ² = 62.6, p = 0.000). Symptom duration of FD lasted less than six months for 70 participants and between six months to one year for 100 participants according to the study results. The sample data showed significant correlations between age factors and symptom durations, affecting gender distribution and psychological diagnoses.

**Table 8:** Descriptive Statistics of Demographic Variables (Age, Gender, Psychological Conditions) f: frequency; %: percentage; p: level of significance; p-values calculated using the chi-square test; the significance level is set at p < 0.05.

Variables	f		Gender		p	χ^²^		Psychological conditions		p	χ^²^
		Male	Female	I prefer not to say			Yes	No	I prefer not to say		
Age	-	-	-	-	<0.001	37.6	-	-	-	<0.001	42.9
Under 18 years	38	18	19	1	-	-	22	10	6	-	-
18-25 years	111	44	61	6	-	-	75	29	7	-	-
26-35 years	56	33	15	8	-	-	37	13	6	-	-
36-45 years	59	10	42	7	-	-	16	34	9	-	-
46-55 years	19	5	9	5	-	-	8	5	6	-	-
56 years or above	17	8	7	2			9	3	5		

Table 9 presents information regarding FD symptoms divided by gender and psychological conditions, together with symptom duration encounters. The research shows that gender substantially links FD symptom durations (p = 0.02, χ² = 18.0). Many individuals experiencing functional dyspeptic symptoms lasting from 6 months to 1 year were identified as male (100), while females reported 44 cases, and 46 people chose not to reveal their gender. The individuals displaying 1-3-year symptoms included 61 males and 25 females. The subject group that experienced symptoms over three years received the fewest responses, comprising 26 males and 11 females. The relationship between FD and psychological conditions was statistically significant (p < 0.001, χ² = 62.6). The presence of psychological conditions was highest among those who experienced symptoms between six months to one year (63 “Yes”) as well as those who had symptoms between one to three years (39 “Yes”). The population group experiencing symptoms for 6 months to 1 year demonstrated both the maximum (24 No) and minimum (28 Yes) rates of absence and presence of psychological conditions, respectively. Participants throughout the studied durations exhibited a substantial preference for not revealing information about their psychiatric state.

**Table 9:** Descriptive Statistics of Demographic Variables (Functional Dyspepsia, Gender, Psychological Conditions) f: frequency; %: percentage; p: level of significance p-values calculated using the chi-square test; the significance level is set at p < 0.05.

Variables	f		Gender		p	χ^²^		Psychological conditions		p	χ^²^
		Male	Female	I prefer not to say			Yes	No	I prefer not to say		
Functional dyspepsia symptoms	-	-	-	-	0.02	18.0	-	-	-	<0.001	62.6
Less than 6 months	70	31	34	5	-	-	49	13	8	-	-
6 months-1 year	100	44	46	10	-	-	63	24	13	-	-
1-3 years	61	25	27	9	-	-	39	15	7	-	-
More than 3 years	26	11	12	3	-	-	14	8	4	-	-
I prefer not to say	43	7	34	2	-	-	2	34	7	-	-

## Discussion

This research examined the association between GI symptom severity and anxiety measurement in FD patients, thus advancing understanding of GI and psychological connections. Our results revealed a powerful connection between patient-witnessed anxiety and GI symptoms, which demonstrates a clear psychosomatic relationship. Research confirms that intense anxiety leads to corresponding GI symptoms. Research involving children revealed that pain-predominant functional gastrointestinal disorders (FGIDs) happened more frequently in people with depression alongside anxiety, thus strengthening proof of psychological distress intensifying intestinal symptom severity [13,20]. Our study data established a solid connection between anxiety symptoms and GI problems, thus confirming how psychological challenges make dyspepsia more severe. Research shows FD patients commonly present higher anxiety levels and neuroticism traits that cause a detrimental impact on perceived symptoms and life quality [8].

Our study confirmed that participants under medication management showed higher levels of GI distress and increased anxiety symptoms, which validated previous findings on FD treatment responses. Our investigation did not measure anxiety levels between different FD subtypes because they can display unique symptom profiles affecting psychological well-being differently. Future research needs to examine these subclassification categories to create better personalized therapeutic strategies [21].

The research results presented contrasting data by showing that younger adults suffered worse GI symptoms than older adults, but existing literature reported higher GI disorder rates among older adults. The inconsistency between studies may result from reporting differences or under-reporting by the elderly population, as well as physiological aging effects combined with multiple medication use in this demographic [22]. The research data indicate that age functions as an important determinant for the intensity of anxiety symptoms. The study’s outcomes support previous research, which demonstrates that young people under 18 and 26-35 years old demonstrate more considerable anxiety levels [23].

The research results showed that male participants, together with individuals who did not disclose their gender, experienced more severe GI symptoms than female participants. Previous studies revealed worse symptom burden in women diagnosed with GI disorders. The observational differences might stem from different participating demographic groups, along with variable ways of symptom recognition and reporting [24]. The study results oppose prior research, which demonstrated that FD impacts women more heavily. Men, together with undisclosed participants, displayed more severe symptoms due to sociocultural elements and collecting sample composition [25]. The results of this study contradict previous meta-analytic findings that present older women as having higher rates of anxiety disorders by showing that both male and female participants displayed increased anxiety scores. The analysis of disparities between groups points toward how different background elements, together with gender matters, affect how people in this study group present and disclose their anxiety symptoms [26]. The analysis established GI symptoms as a major indicator of individual anxiety levels. The previous research findings match our study because they confirm the direct link between GI distress and anxiety disorders, which demonstrates how GI symptoms help initiate anxiety [3]. The findings of our study confirmed that dyspepsia severity, together with GI symptoms, serves as a strong predictor of anxiety while supporting previous research, which shows a notable link between dyspepsia and anxiety disorders [27].

Previous research has confirmed that gender, together with age, affects both psychological and physiological symptoms. Research shows age and gender differences like the present results, despite different directions [25,26]. The study results demonstrated that gender played a significant role in determining FD symptom persistence, which confirms previous research about gastric emptying disparities between genders. The results demonstrate how gender differences require additional attention in FD symptom assessment procedures [28].

Limitations

There are various important limitations of this study. First, its cross-sectional design limits our ability to overcome adverse causal relationships between the severity of GI symptoms and levels of anxiety and QoL because the data were collected at a single point in time. Second, the study depends on self-report measures (GSRS, SF-NDI, and GAD-7) that were a source of recall bias and social desirability bias. Participants might under- or overreport their symptoms in memory or anticipation of the perceived expectations. Furthermore, while ideally FD should be diagnosed clinically with confirmation of a normal upper GI endoscopy to exclude organic causes, not all participants received an endoscopy because of practical factors. This limitation creates the possibility for unresolved organic pathology in some of the respondents, which might have affected the results. Furthermore, while ideally FD should be diagnosed clinically with confirmation of a normal upper GI endoscopy to exclude organic causes, not all participants received an endoscopy because of practical factors. This limitation creates the possibility for unresolved organic pathology in some of the respondents, which might have affected the results. In addition, respondents were limited to a small geographical area of Pakistan, and therefore, its results may not apply to populations of different cultural or socioeconomic backgrounds. Lastly, although GAD-7 is a validated screening instrument, it cannot take the place of formal psychiatric evaluation. There was no formal psychiatric interview (Mini-International Neuropsychiatric Interview (MINI), etc.), and no behavior or therapeutic interventions were explored. Systematic exclusion of psychiatric comorbidities and no behavioral intervention were not assessed. These elements need to be addressed in further studies to create a more detailed picture of the contribution of psychiatric factors to FD.

Future directions

Researchers must conduct longitudinal research to establish how GI symptoms and anxiety levels affect FD patients. Research studies on this topic would show how GI symptoms develop psychological symptoms, along with determining if psychological factors enhance GI conditions. The research would benefit from examining how GI therapy works when combined with psychological treatments that include cognitive behavioral therapy (CBT) or gut-directed hypnotherapy. Studies should study individuals from various backgrounds because they need to understand how cultural factors interact with socioeconomic conditions and demographics regarding the gut-brain axis in FD patients. The study of biological markers together with digestive symptoms and anxiety physiological processes would facilitate improved treatment plans.

## Conclusions

This research has found a relationship between the degree of GI symptom severity and the level of anxiety among FD patients diagnosed based on the Rome IV criteria. Such findings highlight the complex interdependence between physical and psychological well-being in FD patients. Nevertheless, because of the study design and lack of standardized psychiatric evaluation, our findings remain restricted to association and cannot conclude causality. The high levels of anxiety, especially in young adults and those who need medication, underscore the need for early psychological screening and intervention. The causal factors of FD must be addressed in future studies using structured psychiatric interviews, extensive mental status examination (MSE), and investigation of behavioral treatments. In addition, research should create culturally tailored treatment plans that treat both the physical and psychological aspects of FD, which can result in better patient outcomes and QoL.
